# The Optimum Dietary Phenylalanine Requirement of Hybrid Grouper (*Epinephelusfuscoguttatus* ♀ × *Epinepheluslanceolatus* ♂) Juveniles: Effects on Growth Performance, Gut Micromorphology, and Antioxidation

**DOI:** 10.1155/2023/9155290

**Published:** 2023-07-20

**Authors:** Pinxian Yang, Haijiao Wang, Lei Ma, Haoran Yin, Zhanying Zhu, Cong Liu, Wei Huang, Zhiyu Zhou, Xiaoyi Wu, Sehrish Taj

**Affiliations:** ^1^State Key Laboratory of Marine Resource Utilization in South China Sea, Haikou 570228, China; ^2^Hainan Provincial Key Laboratory for Tropical Hydrobiology and Biotechnology, Department of Aquaculture, Hainan University, Haikou, Hainan 570228, China; ^3^Animal Feed Science Research Institute, New Hope Liuhe Co. Ltd, Chengdu, China; ^4^Huzhou Haihuang Biotechnology Co. Ltd, Huzhou, China

## Abstract

The optimum phenylalanine (Phe) requirement for hybrid grouper (*Epinephelusfuscoguttatus* ♀ × *Epinepheluslanceolatus* ♂) juveniles was determined through an 8-week growth trial. A total of seven isoenergetic (340 kcal per 100 g of dry matter), isonitrogenous, and isolipidic diets were made, containing 8.2 (Phe 8.2), 9.2 (Phe 9.2), 10.1 (Phe 10.1), 11.2 (Phe 11.2), 13.3 (Phe 13.3), 15.2 (Phe 15.2), and 17.3 g/kg (Phe 17.3), respectively. Triplicate tanks of juvenile fish (about 16.7 g/fish) were fed each experimental diet twice daily until apparent satiation. The results indicated that different dietary Phe levels significantly influenced weight gain percentage (WG), feed efficiency (FE), protein efficiency ratio (PER), as well as, productive protein value (PPV). Fish fed Phe 8.2 had the lowest WG or PPV among all experimental treatments. Furthermore, the optimal dietary Phe level increased fold height, width, enterocyte, and microvillus height of fish. The Phe 10.1 group exhibited higher growth hormone (GH) expression in the pituitary compared to other groups. Expression of hepatic insulin-like growth factor-1 (IGF-1) and growth hormone receptor 1 (GHR1) displayed a similar pattern of variation to that of GH. The Phe 13.3 group had lower expression of S6 kinase 1 (S6K1) and target of rapamycin (TOR) than other groups. In addition, fish fed Phe 10.1 had lower levels of nuclear factor erythroid 2 (Nrf2) and heat shock protein 70 (HSP70) in the head kidney, and Cu/Zn-superoxide (Cu/ZnSOD) dismutases in the midgut compared to fish fed other Phe levels. Generally, optimal Phe content in the diet of hybrid grouper was estimated to be 12.7 g/kg of dry matter (27.3 g/kg of dietary protein), and at this level, the feed utilization, gut micromorphology, and immunity of fish were also elevated.

## 1. Introduction

Phenylalanine (Phe) is an essential amino acid (EAA) for the protein synthesis, and it is vital for the physiology, metabolism, and protein synthesis in fish [1]. In addition, Phe belongs to aromatic amino acids, and it is important for the metamorphosis of fish larve [2]. Moreover, as a precursor to tyrosine (Tyr), Phe could be converted into epinephrine, norepinephrine, thyroid hormone, triiodothyronine, and thyroxine both in liver and in kidney [1, 3], promoting the growth, tissue differentiation, nutrient metabolism, and antistress capability of fish [3].

The functions of Phe in fish are correlated with its supplemented levels in the diets. Proper Phe supplementations in the diet could positively alter the growth perfomance of various farmed fish species, including rainbow trout (*Oncorhynchus mykiss*) [4], silver perch (*Bidyanusbidyanus*) [5], red drum (*Sciaenops ocellatusto*) [6], Catla (*Catlacatla*) [7], fingerling Nile tilapia (*Oreochromis niloticus*) [8], juvenile blunt snout bream (*Megalobrama amblycephala*) [9], and Indian major carp (*Cirrhinusmrigala*) [10]. As one of the ketogenic amino acids, Phe can elevate the protein and lipid retentions inmany aquatic animals, such as silver perch [5], red drum [6], juvenile grass carp (*Ctenopharyngodon idella*) [1], and juvenile hybrid tilapia (*Oreochromis niloticus × Oreochromis aureus*) [11]. In addition, the appropriate inclusion of Phe to diets could also improve immune status [12], absorptive ability [11], and antioxidant capacity [13] of fish.

Phenylalanine can regulate the transcription levels of fish growth hormone (GH), insulin like growth factor-I (IGF-1), growth hormone receptor 1 (GHR1), target of rapamycin (TOR), and ribosomal protein S6 kinase 1 (S6K1) [13]. Previous research showed that GH and IGF-1 link the nutritional condition and the growth regulation in teleost [14], being as the main genes in the somatotropic axis [15], so the transcriptional level of IGF-1 can be used as an indicator of fish growing [16]. Meanwhile, S6K1 can stimulate the protein synthesis in a variety of cell types [17, 18].

However, both Phe deficiency and excess have adverse effects on growth and health of animals [7, 9]. This means that the optimal dietary Phe requirement should be studied for each farmed fish species. Up to now, it can be found from the published papers that the optimal Phe amounts in diets of farmed fish species range approximately from 4.7 to 36.6 g/kg for different fish species, such as grass carp (initial body weight 3.6 g; 17.6 g/kg Phe of the diet) [1], channel catfish (*ictalurus punctatus*) (195–205 g; 4.7–4.9 g/kg) [19], blunt snout bream (10.28 g; 9.3–10.1 g/kg) [9], Atlantic salmon (*Salmo salar*) (0.2–1500 g; 9.0–16.0 g/kg) [20], red drum (initial weight 1.38 g; 16.9 g/kg) [6], Nile tilapia (*Oreochromis niloticus*) (5.62 g; 11.7–12.1 g/kg) [21], silver perch (1.2 g; 22.0 g/kg) [5], and Catla (0.66 g; 11.2 g/kg) [7].

Hybrid grouper belongs to the marine fish species and has high economic value and disease resistance. Some basic nutritional information [22–24] and the requirements for leucine [25], isoleucine [26], methionine [27], and arginine [28] have previously been reported in our previous studies. However, as one of the EAAs, Phe is still unknown in terms of the nutritional information of hybrid grouper. Therefore, this study aimed to determine the optimal amount of Phe in the diet of hybrid grouper, and hence to evaluate the influence of different Phe levels on growth performance and health of this fish species.

## 2. Materials and Methods

### 2.1. Experimental Diets

The experiment diets (Table 1) were designed to have same crude protein (CP, 464.7 g/kg), crude lipid (CL, 70 g/kg), and gross energy (340 kcal/100 g dry matter), referring to previous studies [26–28, 30, 31]. The overall dietary amino acid contents (Table 2) were adjusted accordingly based on our researches on the reference amino acid profile [24] and the optimum dietary requirements for leucine [25], methionine [27], arginine [28], and threonine [30]. Alanine was added as the substitute in diets with low Phe. Dietary Phe contents were measured to be 8.2, 9.2, 10.1, 11.2, 13.3, 15.2, and 17.3 g/kg dry matter, being abbreviated Phe 8.2, Phe 9.2, Phe 10.1, Phe 11.2, Phe 13.3, Phe 15.2, and Phe 17.3.

The crystalline amino acids (CAAs) in each diet were first weighed and precoated with ceramic matrix composite (CMC) (10 g) and cellulose, and then rubbed by hand to make a powder. The well-ground fishmeal was used during the preparation, along with careful weighing of all the dry ingredients. Dietary preparation was conducted according to the reference methods in our previous study [30]. The bound CAAs was mixed with other dry ingredients and recoated with 229.6–233.7 g corn starch and another 10 g CMC for every 1,000 g dry matter, and then mixed with lipids and water for pelletization. Finally, the diets were air dried in a 25°C room, and the dried diets were then stored at −20°C.

### 2.2. Fish Culturing

Hybrid grouper juveniles from a local aquaculture hatchery (Danzhou Hainan, China) were first given a medicated bath (potassium permanganate and fresh water, 0.5 mg/L, 3 min) to kill the possible pathogenic bacteria and ectoparasites, and then, they were fed a commercial diet (CP and CL contents: 500 and 70 g/kg of dry matter) for 2 weeks to acclimate the water-recycling system which had 21 glass tanks (60 × 45 × 50 cm). After the acclimation, vigorous hybrid grouper juveniles with similar sizes (16.7 ± 0.05 g) were randomly selected as the experimental fish, and each tank had 12 fish. The fish were fed by hand on 08:00 and 16:00, respectively until they were apparently satiated, and the leftover diets were recorded and collected. Each group was given three replicates for 8 weeks and weighed weekly. Seawater quality was daily monitored. The water temperature and oxygen content varied at 29 ± 1°C and 6.1 ± 0.2 mg/L, and and total ammonia nitrogen was below 0.20 mg/L.

### 2.3. Fish Sampling and the Analysis

Prior to the growth trial, 10 fish were randomly collected for measuring initial whole-body CP content. At the end of the feeding, fish were conducted with a 24 hr starvation, and then, they were counted and bulkily weighed. Being same as our previous report [32], fishes were euthanized with MS-222 (Sigma–Aldrich, Germany), and two fish of each tank were randomly sampled for the measurement of the final whole-body proximate compositions. Another three fish taken from each tank were measured the body weight and length individually for calculating condition factor (CF), and then, the blood from caudal vein was obtained by using 1-mL heparinized (Shanghai Pharma, China) syringes. Finally, the dissection was undertaken to achieve the liver and intraperitoneal fat samples which were also separately weighed to calculate viscerosomatic index (VSI), hepatosomatic index (HSI) as well as intraperitoneal fat ratio (IPF). The serum was gotten by centrifuging the blood at 4°C for 10 min (3,000 r/min). Muscle samples were taken from both sides of the body and stored at −20°C. Hypothalamus, head kidney, liver as well as, serum samples were frozen in liquid nitrogen and stored at 80°C. The foregut and midgut samples were taken for the histological analysis.

The moisture content was analyzed by drying samples (at 125°C) to a constant weight using an oven (BGZ-140, China), and the above dried samples were ground in a SI-M81 mixer (Jiuyang, China). CP and CL contents were established by the Dumas combustion method (Elementar, Germany), and the petroleum ether extraction method (ANKOM, America) [28].

### 2.4. Histological Examination of the Foregut and Midgut

Anterior intestines with a length of 1 cm were immersed into Bouin's solution for 24 hr. The intestines were then transferred to 100% ethyl ethanol. The tissues were diluted and dehydrated with different concentrations of alcohol and embedded in paraffin. Embedded tissues were cut into sections of 5 mm, stained with hematoxylin–eosin and then sealed with neutral gum. The Image-Pro Plus 7.0 image analysis software was used. In addition, tissues were analyzed for morphological structures of fold height (hF), fold width (wF), enterocyte height (hE), and microvillus height (hMV) under a light microscope (Olympus IX71).

### 2.5. Total RNA Extraction and Reverse Transcription

Total RNA were extracted from liver, head kidney, and pituitary using Trizol Reagent (Invitrogen, America) and reversely transcribed according to the reference methods described in our previous research [30]. The RNA quality was evaluated through an electrophoresis gel imaging system (Bio-Rad, America). Yield determination was performed using NanoDrop 2000c (Thermo, America). An optical density ratio of 1.8–2.0 was observed between 260 and 280 according to the electrophoresis bands, and luminance detection integrity of total RNA was observed at 28, 18, and 5 s. Following the manufacturer's instructions, reverse transcription reaction (Takara, Japan) and RT–qPCR were performed (Quant Studio 6 Flex, Applied Biosystems, Singapore) [30].

### 2.6. Differentially Expressed Genes Verification Using Quantitative Real-Time Quantitative PCR

Primer pairs used for gene expression analysis were designed by Primer Premier 5.0 based on the nucleotide sequences of IGF-1, TOR, S6K1, GH, GHR1, nuclear factor erythroid 2-(NF-E2-) related factor 2 (Nrf2), Kelch like ECH associated protein 1 (Keap1), heat shock protein 70 (HSP70), Cu/znSOD, tumor necrosis factor-*α* (TNF-*α*), and *β*-actin were used for designing primers (*Supplementary 1*). The standard curve was used to verify the correct PCR amplification efficiency of each primer. The expression level of target gene was calculated by 2^−*ΔΔ*^CT method [33].

### 2.7. Statistic Analysis

Normality and homoscedasticity assumptions were confirmed before any statistical analysis. Based on graded Phe levels, regression analysis of orthogonal polynomial contrasts was used with SPSS 18.0, and the difference was significant at *P* values (significance levels applied) below 0.05. The adjusted *R*^2^ (Adj. *R*^2^) was gotten according to the description of Kvalseth [34]. The optimum dietary Phe requirement based on WG% or PPV was established through a quadratic regression model.

## 3. Results

### 3.1. Growth Performance

**Figure 1:**
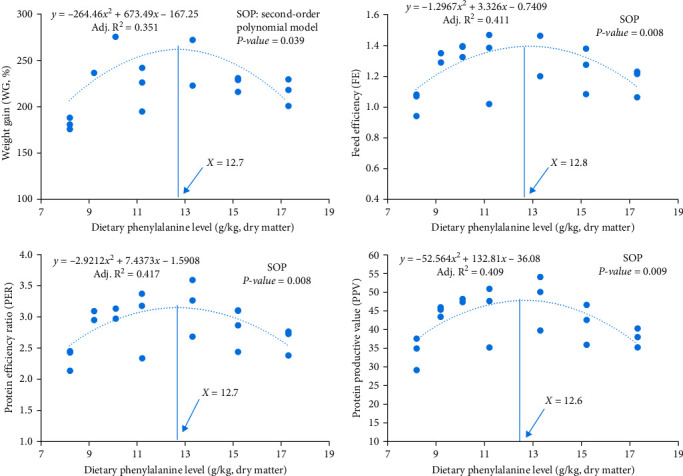
Quadratic regression analysis of WG, FE, PER, and PPV with dietary Phe levels.

No significant differences existed in survival rate among all treatments (*P* ≥ 0.05) (Table 3). The WG first increased in parallel with increasing amounts of dietary Phe with the maximal value being achieved at 10.1 g/kg, and then it decreased at higher Phe amounts. The Phe 8.2 treatment had the lowest PPV, PER, and FE but the highest DFI among all treatments. The quadratic regression analysis of WG or FE against dietary Phe contents indicated that the optimal amount of dietary Phe for hybrid grouper was 12.7 or 12.8 g/kg (Figure 1).

### 3.2. Chemical Composition of Fish and Body Condition Indices

Fish fed Phe 8.2 and Phe 9.2 showed higher HSI or IPF than other Phe fed fish (*P* < 0.05) (Table 4). In addition, different amounts of dietary Phe significantly affected the protein and lipid contents of whole body and muscle (*P* < 0.05) (Table 4). There was no significant difference in CF and whole-body moisture content among different Phe treatment groups.

### 3.3. The Relative mRNA Levels of Genes in Pituitary and Liver

**Figure 2:**
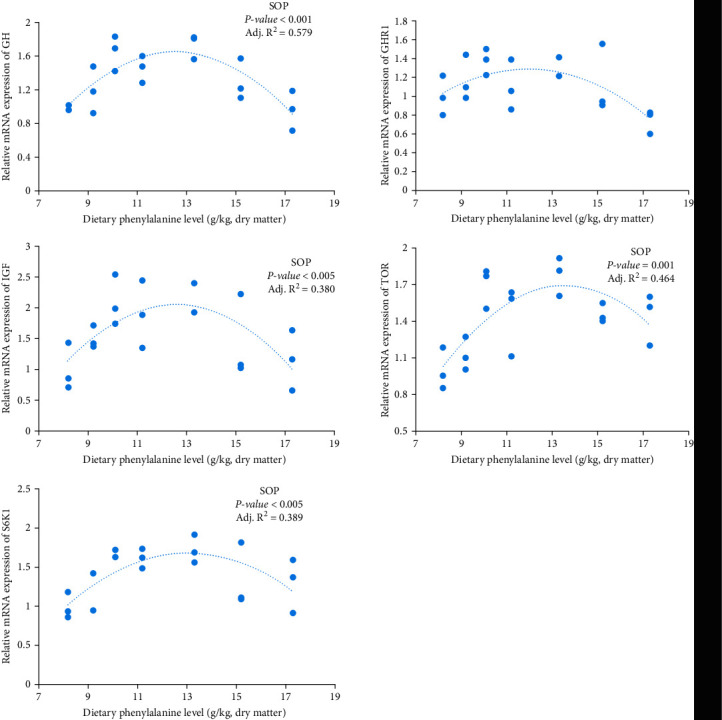
Expression of GH in pituitary and GHR1, IGF-1, TOR, and S6K1 inliver with different dietary Phe levels.

**Figure 3:**
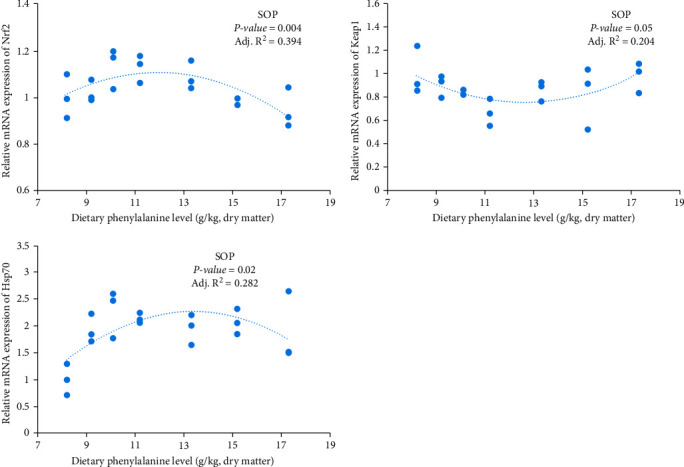
Expression of Keap1, Nrf2, and HSP70 in head kidney of hybrid grouper with different dietary Phe levels.

Compared with other groups, fish fed with Phe 10.1 showed the highest expression of *IGF-1*, *GHR1*, and *GH*, and fish fed with Phe 13.3 had a higher expression of *TOR* and *S6K1*s (Figure 2). Fish fed with Phe 10.1 had the highest mRNA level of *Nrf2* and *HSP70* among all groups, while fishes fed with Phe 11.2 showed the lowest *Keap1* expression (Figure 3).

### 3.4. The Histology of Anterior Gut and Expression of Cu/Zn SOD and TNF-*α* in Midgut

**Figure 4:**
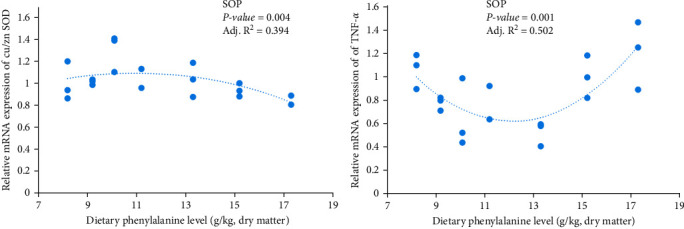
Expression of Cu/Zn SOD and TNF-*α* in midgut with different dietary Phe levels.

Micromorphometric measurements of the fish gut are summarized in Table 5. The wF, hF and hMV values of midgut in Phe 11.2 and 13.3 groups were higher than other groups. Moreover, all groups displayed no significant differences in midgut hE. Among all experimental groups, fish fed Phe 10.1 and Phe 13.3 had the higher hMV, wF, hE, and hF of the foregut. Expression of the TNF-*α* and Cu/Zn *SOD* in the midgut was also significantly affected by different treatments (Figure 4). Compared with other groups, Phe 10.1 had the higher expression of Cu/Zn *SOD* but lower expression of TNF-*α*.

## 4. Discussion

The growth performance and protein absorption of aquatic animals can be improved by suitable dietary Phe supplementations [35], and the Phe requirement of fish usually ranges from 4.7 g/kg to 36.6 g/kg [36]. Among carnivorous fish species, different breeds and specifications have different Phe requirements in their feeds. For example, it was 18.9, 17.6, or 16.0 g/kg Phe for black eel (*Anguilla rostrata*), elver, or Japanese eel (*Anguilla Japonica*) [37]. The Phe demands of different rainbow trout and Atlantic salmon of different specifications were 8.5–16.0 and 11.0–17.0 g/kg [20]. The present research showed that the optimal level of dietary Phe for best growth of hybrid grouper was 10.1 g/kg, being similar to that determined for young grass carp (10.4 g/kg) [18] and juvenile blunt snout bream (9.3–10.1 g/kg) [9], but higher than the value for channel catfish (4.9 g/kg) [19] and juvenile hybrid tilapia (8.78 g/kg) [11]. On the other hand, the amount of dietary Phe required for hybrid grouper was lower than that of silver perch (22.0 g/kg) [5], Catla (11.2 g/kg) [7], or Indian major carp (13.0 g/kg) [10]. The different Phe requirements above may be due to differences in species, ages, experimental conditions, breeding environments, or dietary protein sources.

Fish fed insufficient or excessive Phe had lower WG%, which in turn induced the low FE [13]. At the present study, juvenile hybrid groupers fed a Phe-deficient diet (8.2 g/kg Phe) or a Phe-excessive diet (13.3 g/kg Phe) exhibited significantly poorer values for WG, FE, PER, and PPV compared to other diets. Similarly, previous studies on rainbow trout [4], silver perch [5], grass carp [18], and juvenile blunt snout bream [9] also demonstrated that inadequate or excessive Phe intake reduced the utilization of other amino acids and hence fish growth. The lower PER and PPV of fish fed low Phe perhaps were due to the imbalance of EAAs. On the other hand, the amino acid absorption and utilization will be out of sync in case of an excessive intake of amino acids, resulting in abnormal protein utilization of fish.

Studies on nutrition also focus on the whole-body composition of fish since they have a direct impact on product quality [38]. In this research, the contents of protein and lipid in whole body increased with increasing dietary Phe content up to 10.1 g/kg and then exhibited a declining trend. Meanwhile, fish fed Phe 13.3 were found to have the highest lipid content in the flesh. As dietary Phe content (i.e., a ketogenic amino acid) increased, more Phe were decomposed to provide a carbon skeleton for lipid synthesis, leading to an increase in whole-body CL content [11]. Similar results were observed in rainbow trout [4]. Other authors found that whole-body CP and CL contents tended to increase, while whole-body moisture contents tended to decrease when an optimal level of the Phe was added to the diets of Indian major carp [10], juvenile hybrid tilapia [11], red drum [6], and blunt snout bream [9].

It has been shown that the growth of fish is positively correlated with the expression of GH-IGF axis genes [39]. GH and IGFs mainly control growth, and it has been noted that administration of GH is effective in stimulating IGF-1 production and subsequently fish growth [40, 41]. In this study, fish fed Phe 10.1 exhibited the highest mRNA levels of pituitary GH and liver IGF-1 among all groups, suggesting that the WG variations might be partially due to differential expression of these two genes. This is consistent with our previous report [12]. IGF-1 boosts protein synthesis via the TOR pathway and ribosomal S6K1. S6K1 is a key serine/threonine kinase downstream of the TOR pathway, being required for eukaryotic translation initiation factor 4E (eIF4E) pathways in animals. IGF-1 directly phosphorylates S6K1, which activates S6K1 to promote eIF4E and protein synthesis [42]. Fish fed Phe 13.3 had higher S6K1, TOR expression and PPV than those of other groups, which suggested that the protein synthesis and protein efficiency ratio of fish were stimulated through the TOR/S6K1 signaling pathway.

Gut morphology can be used an indicator for evaluating the utilization of nutrients and fish growth performance [43, 44]. Appropriate levels of dietary amino acids can maintain intestinal health and prevent intestinal diseases [6]. Diets lacking Phe were reported to inhibit the growth of grass carp juveniles, and impair their digestion and absorption [3]. In addition, the Cu/Zn SOD expression in the midgut of the Phe 10.1 group was higher than that obtained other Phe groups, suggesting that appropriate Phe level may increase the antioxidant capacity of the gut. Dietary Phe can promote the intestinal development and increase intestinal health by elevating intestinal mucosal immunity and physical barrier function in aquatic animals [3]. Fish fed Phe 10.1 exhibited the highest wF, hE, and hMV, while fish fed Phe 13.3 displayed the highest hF of the foregut and hMV of the midgut. This may partly explain the growth and feed utilization variations observed in present study.

Intestinal mucosal immunity is closely related to the cytokine-mediated inflammation [45, 46]. TNF-*α* is involved in the systemic inflammation in vertebrates as a proinflammatory cytokine, which resulting in various pathological and functional damages together with other inflammatory factors [8]. In the present study, both excess or deficient Phe content in the diet increased the TNF-*α* mRNA level, while the minimum value existed in the fish fed 13.3 g/kg Phe. Similarly, Feng et al. [13] found that grass carp fed a Phe-deficient diet (8 g/kg) had high mRNA levels of proinflammatory cytokines TNF-*α* and IL-8 in the intestine, whereas the TNF-*α* expression was low in fish provided with optimal dietary Phe. This meant that the optimum dietary Phe level attenuated inflammation and increased intestinal antinutrient capacity, while excessive or deficient dietary Phe aggravated inflammation in juvenile hybrid grouper.

In conclusion, the optimal Phe supplementation recommended for the maximum growth of juvenile hybrid grouper was estimated to be 12.7 g/kg dry matter (27.3 g/kg dietary protein). In addition, insufficient or excess Phe in the diet would depress growth, gut morphology as well as immunity of juvenile hybrid grouper.

## Figures and Tables

**Table 1:** Formulations and proximate composition of experimental diets (dry-matter basis).

Ingredients	Dietary Phe levels (g/kg)						
	8.2	9.2	10.1	11.2	13.3	15.2	17.3
Peruvian fishmeal (anchovy)^a^	210	210	210	210	210	210	210
Poutry byproduct meal^b^	50	50	50	50	50	50	50
Amino acid mixture^c^	255	255	255	255	255	255	255
L-phenylalanine	0	1.5	3	4.5	6	7.5	9
L-alanine	29.2	28.4	27.6	26.8	25.9	25.1	24.3
Chile fish oil (salmon)	42.5	42.5	42.5	42.5	42.5	42.5	42.5
Corn starch	233.7	233	232.3	231.6	231	230.3	229.6
Vitamin mixture^d^	10	10	10	10	10	10	10
Mineral mixture^e^	5	5	5	5	5	5	5
Cellulose	144.6	144.6	144.6	144.6	144.6	144.6	144.6
Carboxymethyl cellulose	20	20	20	20	20	20	20
Analyzed composition							
Dry matter (g/kg)	911.9	921.7	917	913.7	929.4	928.4	914.7
CP (g/kg)	441	435.8	444.5	435.4	447.7	444.5	446.2
CL (g/kg)	67	67	67	67	67	67	68
Phe (g/kg)	8.2	9.2	10.1	11.2	13.3	15.2	17.3

^a^The ingredients were purchased from the Yongsheng Feed Corporation (Binzhou, China), and the proximate composition are as follows: moisture, 85.5; CP, 706.9; CL, 95.8. Amino acid composition (21 g Peruvian fishmeal): lysine 12.2; arginine 10.4; methionine 4.4; threonine 7.1; isoleucine 6.9; leucine 11.6; phenylalanine 6.7; valine 7.6; histidine 6.1; aspartic acid 14.3; serine 6.2; glutamic acid 20.2; glycine 11.2; alanine 9.7; cystine 1.1; tyrosine 5.4; proline 7.1. ^b^Poutry byproduct meal (American Proteins Inc., USA): moisture, 39.9; CP, 641.4; CL, 146.7. Amino acid composition (5 g poutry byproduct meal): lysine 2.1; arginine 2.4; methionine 0.6; threonine 1.4; isoleucine 1.3; leucine 2.4; phenylalanine 1.4; valine 1.6; histidine 0.9; aspartic acid 2.8; serine 1.5; glutamic acid 4.6; glycine 3.2; alanine 2.4; cystine 0.4; tyrosine 0.9; proline 2.2. ^c^Amino acid mixture(g/kg): arginine,23.7; methionine, 9.5; threonine, 3.0; isoleucine, 11.7; leucine, 18.4; valine, 6.5; histidine, 5.9; aspartic acid, 34.4; serine, 14.2; glumatic acid, 66.7; glycine, 17.4; cystine, 5.5; tyrosine, 11.1; proline, 13.9; lysine sulfate (550 g/kg): 13.2. ^d^Vitamin mixture and ^e^mineral mixture see [29].

**Table 2:** Amino acid compositions (g/kg) of experimental diets (dry-matter basis).

AA/∑AA	Dietary Phe levels (g/kg)						
	8.2	9.2	10.1	11.2	13.3	15.2	17.3
EAA							
Lysine	20.2	20.6	19.9	19.4	20.3	20.1	18.6
Arginine	33.9	31.8	32.6	33.2	32.3	32.7	31.8
Methionine	14.3	13.8	13.7	14.2	13.5	13.9	13.8
Threonine	11.1	10.7	11.2	10.5	10.6	11.4	10.8
Isoleucine	19.9	19.4	18.7	18.1	19	19.3	19.9
Leucine	31.8	30.6	30.3	29.3	30.7	30.7	30.8
Phenylalanine	8.2	9.2	10.1	11.2	13.3	15.2	17.3
Valine	15.7	15.7	15.7	14.3	15.3	15.1	14.5
Histidine	11	10	11	10.8	10.8	10.4	10.6
∑EAA	166.1	161.8	163.2	161.0	165.8	168.8	168.1
NEAA							
Aspartic acid	45.1	48.5	48.7	45.9	49.5	49.3	42.5
Serine	19.6	19.1	19.4	19	19.2	19.5	19
Glutamic acid	84.6	81.9	84.4	87.8	83.9	81.8	86.6
Glycine	28.1	25.6	25.9	26.5	27.2	27.2	30.3
Alanine	38.4	36.1	32.8	33.1	34	35.7	34.1
Cystine	5.8	2.8	2.9	1.4	5.9	3.9	4.4
Tyrosine	16.5	15.3	15.2	15	15.2	17.1	16
Proline	24.3	22.8	22.4	21.8	22.6	23.3	24.4
∑NEAA	262.4	252.1	251.7	250.5	258.6	257.8	257.3
∑AA	428.5	413.9	414.9	411.5	423.3	426.6	425.4

**Table 3:** Growth performance and feed utilization of hybrid grouper juveniles fed different dietary Phe levels for 8 weeks.

Dietary Phe levels (g/kg)	WG (%)^a^	DFI^b^ (g/100 g fish/day)	FE^c^	PER^d^	PPV^e^ (%)	Survival (%)
8.2	182	1.74	1.03	2.34	33.93	100
9.2	237	1.37	1.31	3.00	44.89	100
10.1	299	1.31	1.37	3.08	47.61	94
11.2	221	1.42	1.29	2.96	44.57	100
13.3	266	1.27	1.42	3.18	47.96	97
15.2	225	1.45	1.24	2.80	41.74	97
17.3	216	1.53	1.17	2.62	37.84	94
*PSE* ^6^	8.62	0.04	0.04	0.08	1.41	0.92
*Regression* (*N* = 3)						
SOP						
Adj. *R*^2^	0.351	0.401	0.411	0.417	0.409	0.140
*P*-Value	0.039	0.010	0.008	0.008	0.009	0.258

^a^WG%, weight gain; ^b^DFI, daily feed intake; ^c^FE, feed efficiency; ^d^PER, protein efficiency ratio; ^e^PPV, protein productive value; ^f^PSE, pooled standard error of treatment means (*n* = 3).

**Table 4:** Body condition indices; whole-body and white muscle compositions (fresh-weight basis, g/kg) of hybrid grouper juveniles fed different dietary Phe levels for 8 weeks.

Dietary Phe levels (g/kg)	Body condition indices			Whole-body composition^a^			White muscle composition		
	HSI	IPF	CF	Moisture	CP	CL	Moisture	CP	CL
8.2	44	14.4	26.5	754.9	146	42.6	776	190.9	10
9.2	41.9	14.7	30.7	743.1	149.2	47.7	766.1	196	13.7
10.1	36.7	13.8	28.6	744.9	153	54.2	763.6	197.5	16.8
11.2	38.8	13.7	26.5	740.7	149.8	52.5	762	196.1	16.3
13.3	30.9	12.4	27.7	748.2	150	49.9	759.7	197.7	15.6
15.2	36.3	13.5	27.5	740.1	148.7	49.8	760.4	193.8	15.2
17.3	42.2	13.8	26.7	745.9	145.7	48.2	776.1	189.9	14.4
*PSE*	1.60	0.39	0.99	0.15	0.07	0.10	0.22	0.50	0.05
*Regression* (*N* = 9)									
SOP									
Adj. *R*^2^	0.499	0.244	−0.046	0.181	0.322	0.326	0.411	0.301	0.536
*P*-Value	0.001	0.031	0.583	0.166	0.030	0.029	0.009	0.040	0.001

^a^Initial whole-body composition (g/kg): moisture = 766.5; CP = 147.6; CL = 31.4; HSI, hepatosomatic index; IPF, intraperitoneal fat ratio; CF, condition factor.

**Table 5:** Gut micromorphology of hybrid grouper juveniles fed different dietary Phe levels.

Dietary Phe levels (g/kg)	Foregut				Midgut			
	HF (*μ*m)^a^	WF (*μ*m)^b^	HE (*μ*m)^c^	HMV (*μ*m)^d^	HF (*μ*m)	WF (*μ*m)	HE (*μ*m)	HMV (*μ*m)
8.2	283	60	30	3.73	207	63	30	3.52
9.2	269	67	31	4.07	288	69	33	3.81
10.1	469	79	38	5.41	387	75	37	4.10
11.2	371	70	37	4.38	356	83	41	4.05
13.3	513	74	34	4.22	423	71	33	4.73
15.2	394	74	33	4.33	366	77	32	4.12
17.3	299	64	32	4.03	317	72	35	4.12
*PSE*	20.47	1.53	0.79	0.13	20.62	1.73	0.92	0.11
*Regression* (*N* = 3)								
SOP								
Adj. *R*^2^	0.542	0.537	0.444	0.307	0.426	0.303	0.164	0.380
*P*-Value	0.001	0.001	0.005	0.037	0.007	0.039	0.200	0.013

^a^hF, fold height; ^b^wF, fold width; ^c^hE, enterocyte height; ^d^hMV, microvillus height.

## Data Availability

Data supporting the results in the present study are available from the corresponding author upon legitimate request.

## Supplementary Materials

Supplementary Materials
*Supplementary 1*: Primers used for quantitative RT-PCR (qPCR).
