# Supplementary material for: The Optimum Dietary Phenylalanine Requirement of Hybrid Grouper (Epinephelusfuscoguttatus ♀ × Epinepheluslanceolatus ♂) Juveniles: Effects on Growth Performance, Gut Micromorphology, and Antioxidation
Source: Aquac Nutr. 2023 Jul 20;2023:9155290. doi: 10.1155/2023/9155290 (PMC10374384; doi:10.1155/2023/9155290)
Supplement: Supplementary Materials — Supplementary 1: Primers used for quantitative RT-PCR (qPCR). [file 9155290.f1.docx]

**Supplement Table 1**

Primers used for quantitative RT-PCR (qPCR).

| Used for | | Gene name | Gene bank accession no. | Primer sequence (5’-3’) |
| --- | --- | --- | --- | --- |
| qPCR | | GH^1^ | EU280321.1 | F^11:^ AGAGACTCTTCTCCGACTTTGAG |
|  | |  |  | R^12^: CTCCCAGGACTCCACCAAC |
|  | | GHR1 ^2^ | KR269817.1 | F: CACAGACTTCTATGCCCAGGT |
|  | |  |  | R: GTGTAGCCGCTTCCTTCAG |
|  | | IGF-1^3^ | AY776159.1 | F: TATTTCAGTAAACCAACAGGCTATG |
|  | |  |  | R: TGAATGACTATGTCCAGGTAAAGG |
|  | | TOR^4^ | JN850959.1 | F: TCTCCCTGTCCAGAGGCAATAA |
|  | |  |  | R: CAGTCAGCGGGTAGATCAAAGC |
|  | | S6K1^5^ | XM_020085100.1 | F: TCCTTCTCCGTCTGTAAACGA |
|  | |  |  | R: CATGAACACCTGCTTACCAT |
|  | Cu/Zn SOD^6^ | | AY035854.2 | F: CCTGGTGAGCATGGTTTC |
|  | |  |  | R: TGAGGGTGATTATCTTGTCC |
|  | | TNF-α^7^ | FJ491411.1 | F: GAGGACGGTGGTGTTGGTGG |
|  | |  |  | R: TTCTCTTTGGCCTGATTGCG |
|  | | HSP70^8^ | AY423555.2 | F: GTCCTGATCAAACGAAACACCA |
|  | |  |  | R: CACGCTCACCCTCATAAACCT |
|  | | Keap1^9^ | XM_018665037.1 | F: TCCACAAACCCACCAAAGTAA |
|  | |  |  | R: TCCACCAACAGCGTAGAAAAG |
|  | | Nrf2^10^ | KU892416.1 | F: TATGGAGATGGGTCCTTTGGTG |
|  | |  |  | R: GCTTCTTTTCCTGCGTCTGTTG |
|  | | β-actin | AY510710.2 | F: CTCTGGGCAACGGAACCTCT  R: GTGCGTGACATCAAGGAGAAGC |

^1^GH: growth hormone;

^2^GHR1: growth hormone receptor 1;

^3^IGF-1: insulin like growth factor-1;

^4^TOR: target of rapamycin;

^5^S6K1: ribosomal protein S6 kinase 1;

^6^Cu/Zn SOD: Cu/Zn- superoxide dismutase;

^7^TNF-α: tumor necrosis factor-α;

^8^HSP70: heat shock proteins 70;

^9^Keap1: Kelch sample related protein-1;

^10^Nrf2: NF-E2-related factor 2

^11^F: Forward sequence;

^12^R: Reverse sequence;
